# Evaluation of expanded peripheral blood derived CD34+ cells for the treatment of moderate knee osteoarthritis

**DOI:** 10.3389/fbioe.2023.1150522

**Published:** 2023-05-23

**Authors:** Christine Vignon, Morgane Hilpert, Karine Toupet, Aurélien Goubaud, Danièle Noël, Matthieu de Kalbermatten, Philippe Hénon, Christian Jorgensen, Andrea Barbero, Ibon Garitaonandia

**Affiliations:** ^1^ CellProthera, Mulhouse, France; ^2^ Department of Biomedicine, University Hospital Basel, University of Basel, Basel, Switzerland; ^3^ Institute of Regenerative Medicine and Biotherapy, University of Montpellier, INSERM, Montpellier, France

**Keywords:** osteoarthritis, regenerative medicine, CD34+ cells, ProtheraCytes^®^, chondrocytes

## Abstract

Knee osteoarthritis (OA) is a degenerative joint disease of the knee that results from the progressive loss of articular cartilage. It is most common in the elderly and affects millions of people worldwide, leading to a continuous increase in the number of total knee replacement surgeries. These surgeries improve the patient's physical mobility, but can lead to late infection, loosening of the prosthesis, and persistent pain. We would like to investigate if cell-based therapies can avoid or delay such surgeries in patients with moderate OA by injecting expanded autologous peripheral blood derived CD34+ cells (ProtheraCytes^®^) into the articular joint. In this study we evaluated the survival of ProtheraCytes^®^ when exposed to synovial fluid and their performance *in vitro* with a model consisting of their co-culture with human OA chondrocytes in separate layers of Transwells and *in vivo* with a murine model of OA. Here we show that ProtheraCytes^®^ maintain high viability (>95%) when exposed for up to 96 hours to synovial fluid from OA patients. Additionally, when co-cultured with OA chondrocytes, ProtheraCytes^®^ can modulate the expression of some chondrogenic (collagen II and Sox9) and inflammatory/degrading (IL1β, TNF, and MMP-13) markers at gene or protein levels. Finally, ProtheraCytes^®^ survive after injection into the knee of a collagenase-induced osteoarthritis mouse model, engrafting mainly in the synovial membrane, probably due to the fact that ProtheraCytes^®^ express CD44, a receptor of hyaluronic acid, which is abundantly present in the synovial membrane. This report provides preliminary evidence of the therapeutic potential of CD34+ cells on OA chondrocytes *in vitro* and their survival after *in vivo* implantation in the knee of mice and merits further investigation in future preclinical studies in OA models.

## 1 Introduction

Osteoarthritis (OA) is the most prevalent rheumatic diseases, causing pain, reducing mobility and quality of life (Heidari, 2011). OA is characterized by cartilage degradation, subchondral bone sclerosis, calcification of ligaments and synovial inflammation (Li et al., 2013). The main risks factors are age, obesity, genetics and injuries following traumas (Blagojevic et al., 2010). The prevalence of OA is constantly increasing and enormous public health resources are devoted to the treatment of this disease (Cross et al., 2014). Current treatments are limited to use of antalgics and non-steroid anti-inflammatory drugs to alleviate symptoms and delay end-stage treatment of joint replacement by a prosthesis (da Costa et al., 2017). This irreversible surgical intervention is only considered as the very last choice, largely due to the limited durability of the implant (Rönn et al., 2011).

New therapeutic strategies are being developed and one of these is the use of cell-based therapies. Different cell therapies have been tested for the treatment of knee OA with promising results (Zhao et al., 2018). The great potential of this approach was demonstrated by the autologous chondrocyte implantation, one of the first cell therapy approaches in knee OA (Davies and Kuiper, 2019). More recently, mesenchymal stromal cells (MSCs) are increasingly being considered as a promising alternative as injective approach to target OA (Song et al., 2020). Indeed, MSCs isolated from different sources have been shown to secrete many bioactive trophic factors that exert both immunomodulatory and regenerative actions (Bernardo et al., 2009; Dabrowska et al., 2020). The isolation of stromal cells, is however a surgical strategy that can cause donor site morbidity and infection (Mastrolia et al., 2019).

Additional autologous cell sources are thus under investigations. In particular, ProtheraCytes^®^, an advanced therapeutic medicinal product (ATMP) derived from autologous expanded CD34+ cells can represent a novel source for the treatment of OA cartilage lesion. ProtheraCytes^®^ have regenerative properties and have shown to improve global cardiac function in post-acute myocardial infarction (Saucourt et al., 2019). In a pilot clinical study, we showed that intramyocardial delivery of human CD34+ cells in post-acute myocardial infarction (AMI) patients is safe and leads to long term improvement (Pasquet et al., 2009). Even patients who were initially recommended for a heart transplant, no longer required it several years after the cell injection. Based on these promising clinical results, we are now conducting a multicenter, randomized, controlled Phase 2 clinical trial in severe AMI to investigate the safety and efficacy of Protheracytes^®^ (ClinicalTrials.gov Identifier: NCT02669810). Protheracytes^®^ are manufactured using the StemXpand^®^ system, which is a closed, automated, ISO13485:2016 certified device for GMP manufacturing that we developed.

In this study, we decided to investigate the therapeutic potential of ProtheraCytes^®^ in an *in vitro* OA cartilage model. In this *in vitro* OA model, we cocultured ProtheraCytes^®^ with human OA chondrocytes in medium containing pro-inflammatory cytokines (Acevedo Rua et al., 2021) to investigate their survival and capacity to modulate their inflammatory/degrading state. We also evaluated the survival and engraftment of ProtheraCytes^®^ after intra-articular (IA) injection in the murine model of collagenase-induced osteoarthritis (CIOA) to determine the best timing of cell injection for future therapeutic efficacy studies in this animal model.

## 2 Materials and methods

### 2.1 ProtheraCytes^®^ culture

ProtheraCytes^®^ were obtained after expansion of mobilized CD34+ cells from frozen healthy donors (Lonza, NC, United States) as previously described (Saucourt et al., 2019). ProtheraCytes^®^ were conditioned in 2% human serum albumin (HSA) in phosphate buffered saline (PBS) in sterile prelabelled syringes and shipped the same day in a refrigerated box to the Cartilage Engineering laboratory at the University Hospital Basel for analysis.

### 2.2 Coculture of ProtheraCytes^®^ with OA synovial fluid

ProtheraCytes^®^ resuspended in 2% HSA/PBS were incubated with four different concentrations (0, 10, 20 and 50%) of synovial fluid from six OA patients (donor 1: female, 73 years; donor 2, male, 70 years; donor 3, male, 79 years; donor 4, male, 67 years; donor 5, female, 70 years; donor 6, male, 69 years) and for five different timepoints (1, 3, 6, 24, and 96 h). The synovial fluid samples were provided by Dr. S. Lefebvre, MD from the Mulhouse Institute of the Musculoskeletal System after signed informed consent. Cell number and viability were assessed with the Stem Cell Enumeration kit (BD) via flow cytometry with FACS Canto II and FACSDiva software (BD Biosciences).

### 2.3 Cartilage sample collection

Macroscopically fibrillated human articular cartilage was obtained from the knee joints of 4 donors (donor 1: male, 63 years; donor 2: male, 52 years; donor 3: female, 56 years; donor 4: male, 76 years) with clinical history of OA who were undergoing total knee replacement, after informed consent from patients and in accordance with the Institutional Ethics Committee (University Hospital Basel, Switzerland). A histological image of a portion of the joint specimen from donor 2 from where the cartilage sample was harvested, is depicted in Supplementary Figure S1.

### 2.4 Chondrocyte isolation and expansion

OA chondrocytes were isolated from native tissues after 22 h enzymatic digestion in 1.5 mg/mL collagenase as previously described (Barbero et al., 2004). Isolated cells were resuspended in Complete medium consisting of Dulbecco’s modified Eagle’s medium (DMEM) containing 10% fetal bovine serum (FBS), 10 mM Hepes buffer, 1 mM sodium pyruvate, 100 U/mL penicillin, 100 mg/mL streptomycin, and 0.29 mg/ml L-glutamine (all from Gibco) and seeded at a density of 10,000 cells/cm^2^ and cultured in complete medium supplemented with 1 ng/mL Transforming Growth Factor-β1 (TGF-β1) and 5 ng/mL Fibroblast Growth Factor-2 (FGF-2). When cells were approximately 80% confluent, first passage (P1) cells were detached using 0.05% trypsin/0.53 mM EDTA and frozen in FBS containing 10% dimethylsulfoxide.

### 2.5 Chondrocyte/ProtheraCytes^®^ co-culture experiment

Passage 1 OA chondrocytes were thawed and seeded in 24 well plates at a density of 10,000 cells/cm^2^ and cultured one additional week in complete medium. ProtheraCytes^®^ sent to the Cartilage Engineering laboratory of the University Hospital Basel were centrifuged, counted, and seeded onto 0.4 µm pore size polycarbonate Transwell filters (Corning B.V. Life Science) at a density of 100,000 (0.1 M) and 250,000 (0.25 M) cells/insert. Control groups consisted of only OA chondrocytes (in the well) or ProtheraCytes^®^ (in the insert). In the first experiment with OA chondrocytes from one donor (donor 1) and one ProtheraCytes^®^ batch, cells were co-cultured for 6, 24, and 48 h in complete medium containing 5% FBS in the absence (−INFL) or presence of inflammatory cytokines (+INFL: 50 pg/mL Interleukin (IL)-1β, 100 pg/mL IL-6 and 50 pg/mL tumour necrosis factor (TNFα) (Acevedo Rua et al., 2021). In the second experiment, OA chondrocytes from three additional donors (donor 2–4) and two ProtheraCytes^®^ batches were co-cultured only for 24 h, after this timepoint was selected from the first experiment. Three replicates/group were analysed. At the end of the experiments, supernatants were collected and stored at −80°C for subsequent quantification of cytokines; OA chondrocytes were counted with trypan blue and processed for RT-PCR analyses, and ProtheraCytes^®^ were assessed by flow cytometry.

### 2.6 Flow cytometry analysis

ProtheraCytes^®^ were washed with PBS and stained with 0.1 μg/mL 4′,6-diamidino-2-phenylindole (DAPI) (ThermoFisher) in PBS containing 0.1% bovine serum albumin (BSA) and 2 mM EDTA. ProtheraCytes^®^ were analyzed on the Cytoflex flow analyzer (BD), and the frequency of dead cells was measured.

### 2.7 Quantitative real-time RT-PCR

RNA was extracted from OA chondrocytes using the Quick RNA mini prep kit (Zymo), according to the manufacturer’s instructions. Complementary DNA (cDNA) was generated from the maximum amount of RNA possible for each sample (from 200 to 1,000 ng) by using 500 μg/mL random hexamers (Promega, Madison, WI) and 0.5 μL of 200 units/mL Reverse transcriptase superscript III (Invitrogen), in the presence of dNTPs and DTT. The PCR was based on TaqMan reaction using the TaqMan mix (Thermo Fisher scientific). cDNA samples (2 μL, for a total volume of 10 μL per reaction) were analysed both for gene of interest ACAN (Hs00153936), Col1A1 (Hs00164004), Col2A1 (Hs00264051), Col10A1 (Hs00166657), MMP-1 (Hs00233958), MMP-13 (Hs00233992), SOX9 (Hs00165814), CXCL8 (Hs00174103), IL1β (Hs01555410), IL6 (Hs00985639), ADAMTS-4 (Hs00192708), ADAMTS-5 (Hs00199841), NFkB1 (Hs00765730_m1) and for the housekeeping gene GAPDH (Hs2758991) (all from Applied Biosystems). For each cDNA sample, the threshold cycle (Ct) value of each target sequence was subtracted from the Ct value of the reference gene, to derive ΔCt. The level of expression of each target gene was then calculated as 2^−ΔΔCT^. Each sample was assessed at least in duplicate for each gene of interest.

### 2.8 Quantification of secreted proteins

Supernatants collected after the coculture experiments were centrifuged for 4 min at 16,000 × g to remove cellular debris. The concentrations of IL1β, IL6, IL8/CXCL8, RANTES/CCL5, TNFα and VEGF were quantified by Luminex Magnetic Assay (R&D Systems), while the concentrations of ADAMTS-4 and ADAMTS-5, MMP-1 and MMP-13 were measured using DuoSet ELISA systems, according to manufacturer’s instruction.

### 2.9 *In vitro* assessment of ProtheraCytes^®^ after delivery with a needle for intra-articular injection

We evaluated the viability, purity, and number of ProtheraCytes^®^ before and after delivery with a needle for knee injection in humans (21G, 2″BD301155) and mice (20G, 25 mm BD304827). These parameters were evaluated with the Stem Cell Enumeration kit (BD) via flow cytometry with FACS Canto II and FACSDiva software (BD Biosciences).

### 2.10 Murine model of collagenase-induced osteoarthritis

The collagenase-induced osteoarthritis (CIOA) model was induced by collagenase injection (type VII collagenase from *Clostridium* histolyticum; 1 U in 5 μL saline solution) in the intra-articular space of knee joints of severe combined immunodeficient (SCID) Beige immunodeficient male mice at 8 weeks of age, at day 0 and day 2. This treatment induces ligament laxity and knee instability leading to osteoarthritic like lesions. ProtheraCytes^®^ were injected intra-articularly in the knee joint of mice at day 8 or day 29 after OA induction. ProtheraCytes^®^ were injected at the dose of 250,000 cells in 5 μL of 5% HSA in physiological serum. Mice were euthanised 1, 7 or 21 days after cell injection. Therefore the mice were divided in six groups with four mice per group: Group 1 (collagenase + ProtheraCytes^®^ at day 8, euthanasia at day 9); Group 2 (collagenase + ProtheraCytes^®^ at day 8, euthanasia at day 15); Group 3 (collagenase + ProtheraCytes^®^ at day 8, euthanasia at day 29); Group 4 (collagenase + ProtheraCytes^®^ at day 29, euthanasia at day 30); Group 5 (collagenase + ProtheraCytes^®^ at day 29, euthanasia at day 36); Group 6 (collagenase + ProtheraCytes^®^ at day 29, euthanasia at day 50). Mice were housed in cages with unlimited access to food and water in a specific pathogen-free animal facility under controlled atmosphere. For euthanasia, animals were anesthetized with 3% isoflurane and then submitted to cervical dislocation. At euthanasia, hind paws were collected and fixed in 3.7% formaldehyde for 4 days at ambient temperature. Hind paws were then rinsed twice in PBS and stored at 4°C in PBS until histological processing.

### 2.11 Immunohistochemistry staining of ProtheraCytes^®^

Hind paws were decalcified in TBD-2 solution (Fisher Scientifc) for 24 h at room temperature and then rinsed in PBS before inclusion in paraffin. After paraffin inclusion of samples, three frontal sections of 7 μm in thickness and spaced by 100 μm were recovered from each sample. Sections were then immunostained using the Alu Positive Control Probe II from Ventana (Roche). This Alu probe consists of a cocktail of oligonucleotide probes labelled with dinitrophenol (DNP) that are specific for human DNA. Analysis of immunostaining was performed by attributing an arbitrary score from “−” (absence of immunostaining) to “+++” (strong immunostaining) for the 3 frontal sections of each sample. Additional immunostaining was performed for the detection of CD45^+^ (Abcam, cat# Ab133469) human cells and caspase 3^+^ (Cell signaling, cat# 96615, clone ASP175) apoptotic cells on serial sections. Slides were scanned using the Nanozoomer 2.0 Hamamatsu in order to illustrate the immunostaining scoring.

### 2.12 Statistical analysis

All data are presented as mean values ±SD. Using the statistical analysis software GraphPad Prism, Mann Whitney *U* testing or Kruskal–Wallis for non-parametric unpaired sample sets were performed. For each donor and experimental group, technical duplicates or triplicates were performed. Unilateral *p* values < 0.05 were considered significant.

## 3 Results

### 3.1 OA synovial fluid supports the survival of ProtheraCytes^®^

**FIGURE 1:**
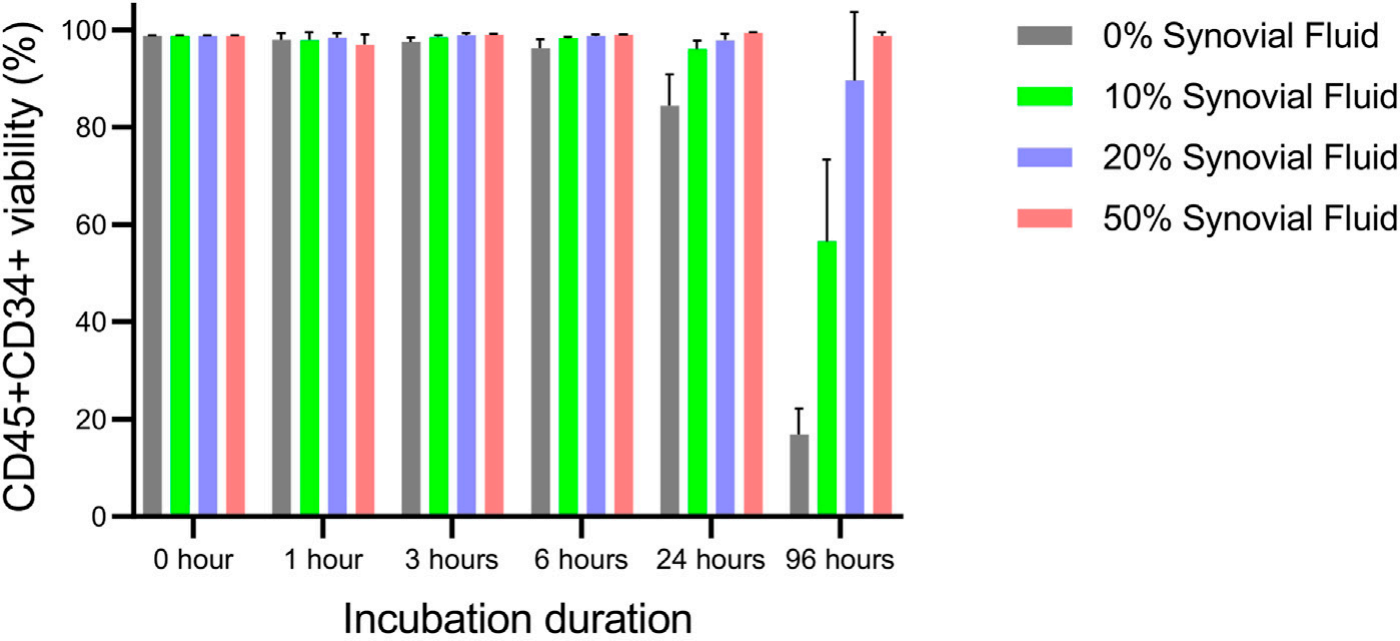
Viability assessment of ProtheraCytes^®^ in OA synovial fluid. The viability of Protheracytes^®^ in 2% HSA/PBS buffer was assessed after incubation at 37°C, 5% CO_2_ with different concentrations (0%, 10%, 20%, 50%) of OA synovial fluid at different timepoints (0, 1, 3, 6, 24, and 96 h). Values are mean ± SD of measurements from 3 replicates.

When Protheracytes^®^ in 2% HSA/PBS buffer were incubated at 37°C, 5% CO_2_ with different concentrations (0, 10, 20, 50%) of OA synovial fluid at different timepoints (0, 1, 3, 6, 24, and 96 h), we observed that cell viability varied little from 99.0% to 96.2% from 0 to 6 h but decreased to 84.4% after 24 h only for the 0% synovial fluid condition (Figure 1). However, at 96 h of incubation, cell viabilities decreased sharply to 16.9% in 0% synovial fluid, 56.6% in 10% synovial fluid, 89.7% in 20% synovial fluid but there was no decrease in the 50% synovial fluid condition with a viability of 98.7% (Figure 1).

### 3.2 ProtheraCytes^®^ can modulate the expression of some inflammatory and pro-anabolic genes in OA chondrocytes

**FIGURE 2:**
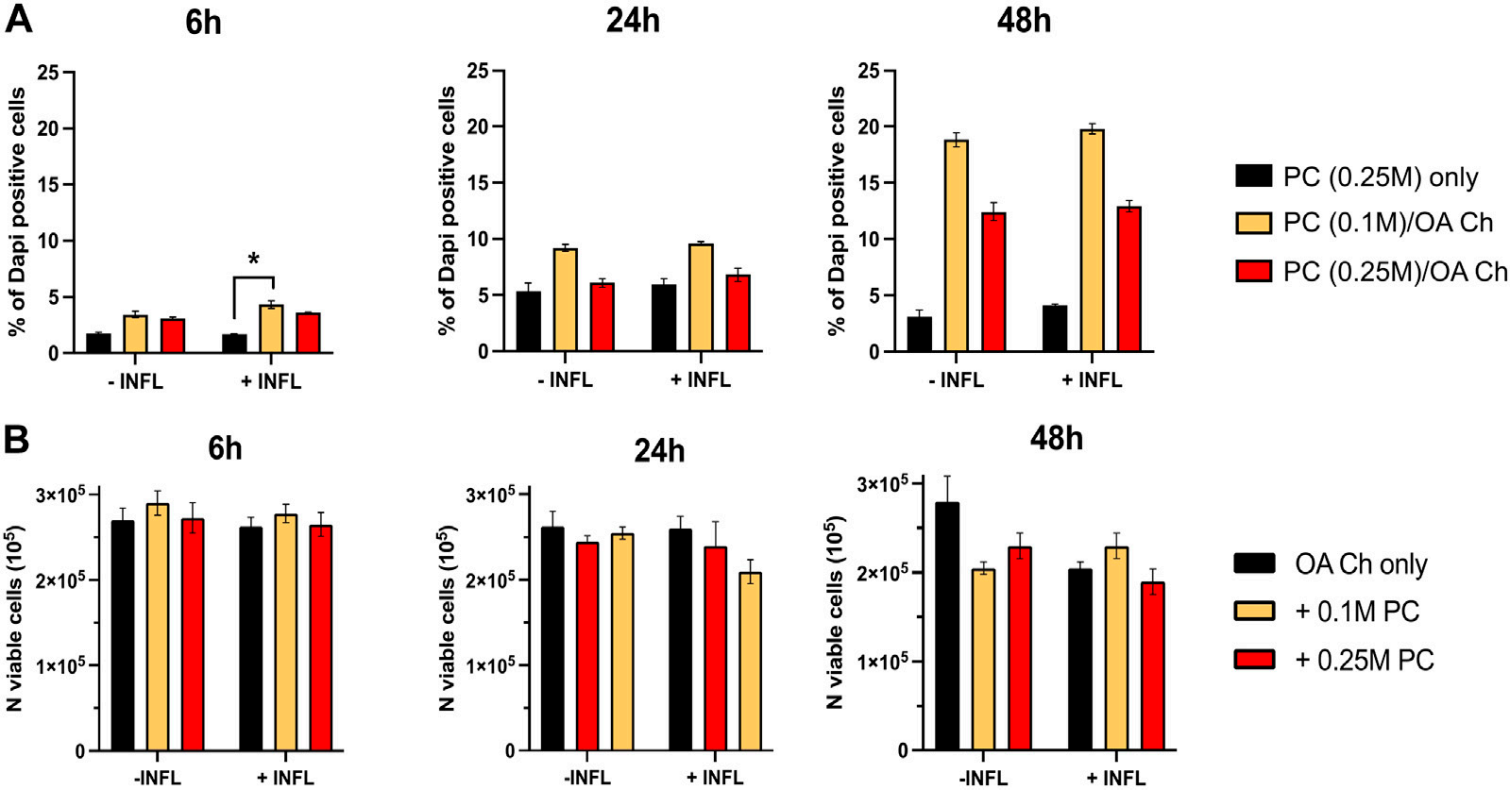
Assessment of ProtheraCytes^®^ and OA chondrocyte survival after coculture at different timepoints. **(A)** Percentage of dead ProtheraCytes^®^ (PC) estimated by flow cytometry by counting the number of PC DAPI positive cells, after 6, 24 and 48 h of monoculture [PC (0.25 M) only] or co-culture with OA chondrocytes at 0.1 or 0.25 M cells/insert [PC (0.1 M)/OA Ch and PC (0.25 M)/OA Ch, respectively] in the absence (−INFL) or presence (+INFL) of inflammatory cytokines. Values are mean ± SD of measurements from 3 replicates *p < 0.05. **(B)** Number of viable OA Chondrocytes after 6, 24 and 48 h of monoculture (OA Ch only) or co-culture with 0.1 or 0.25 M ProtheraCytes^®^/well (+0.1 M ProtheraCytes^®^ and +0.25 M ProtheraCytes^®^, respectively) in the absence (−INFL) or presence (+INFL) of inflammatory cytokines. Values are mean ± SD of measurements from 2 replicates.

To optimize the experimental conditions, we performed a first coculture experiment with OA chondrocytes and different numbers of ProtheraCytes^®^/insert (0, 0.1, 0.25 million cells/insert), different timepoints (6, 24, and 48 h), and in the presence (+INFL) or absence (−INFL) of inflammatory cytokines. We observed that the percentage of dead ProtheraCytes^®^ (PC) was similar in the inflammatory and non-inflammatory conditions and increased from 6 to 24 h (up to 5%) remaining unchanged at 48 h of culture (Figure 2A). The highest cell death was observed when ProtheraCytes^®^ were cocultured at the lowest density (0.1 M cells/insert) with OA chondrocytes (Figure 2A).

We also assessed the number of viable OA chondrocytes and at 6 h, no difference could be observed in any of the co-culture conditions performed in the absence or presence on inflammatory cytokines (Figure 2B). At 24 h, a slight reduction in the number of viable OA chondrocytes cocultured with the highest dose of ProtheraCytes^®^ (0.25 M cells/insert) was observed in the inflammatory condition. At 48 h, the number of viable OA chondrocytes decreased when cocultured with ProtheraCytes^®^ in the absence of inflammatory cytokines (Figure 2B).

**FIGURE 3:**
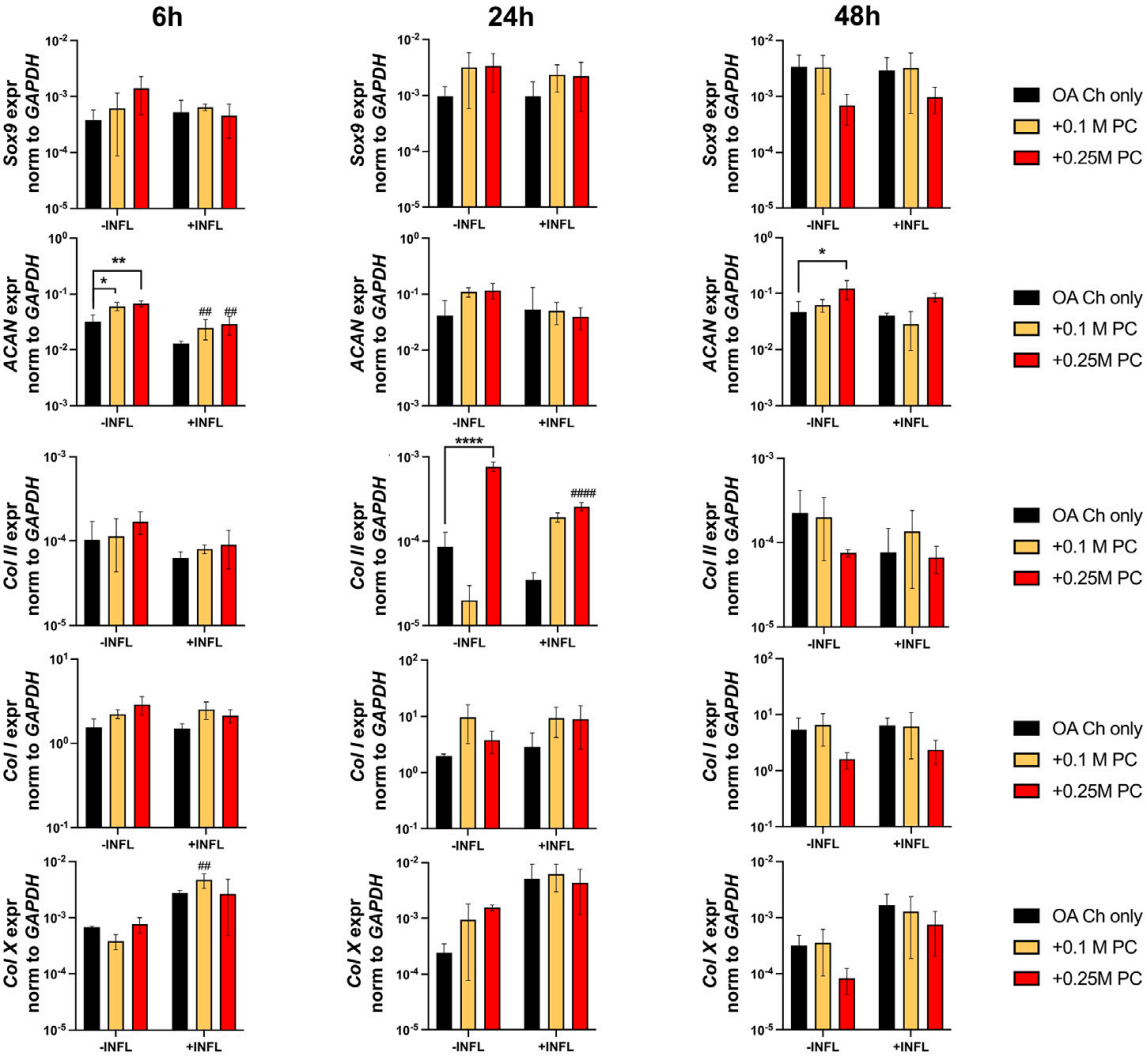
Expression levels of chondrogenic genes in OA chondrocytes at different timepoints. Real-time reverse transcriptase–polymerase chain reaction of OA Chondrocytes after 6, 24 and 48 h of monoculture (OA Ch only) or co-culture with 0.1 or 0.25 M ProtheraCytes^®^/well (+0.1 M PC and +0.25 M PC, respectively) in the absence (−INFL) or presence (+INFL) of inflammatory cytokines. Values are mean ± SD of measurements from 4 independent donors in 3 replicates. * = *p* < 0.05, ** = *p* < 0.01, *** = *p* < 0.001. ^#^ = *p* < 0.05, ^##^ = *p* < 0.01, ^###^ = *p* < 0.001, difference between −INFL and +INFL at identical conditions.

**FIGURE 4:**
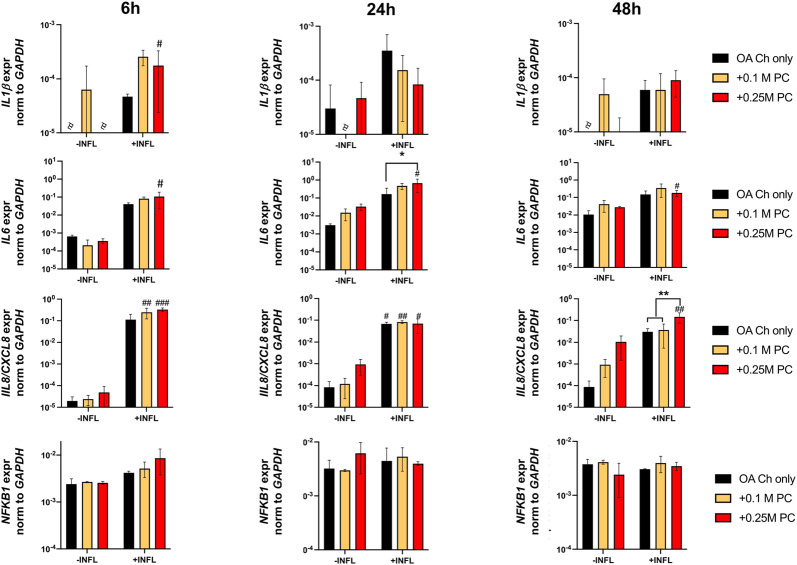
Expression levels of pro-inflammatory genes in OA chondrocytes at different timepoints. Real-time reverse transcriptase–polymerase chain reaction of OA Chondrocytes after 6, 24 and 48 h of monoculture (OA Ch only) or co-culture with 0.1 or 0.25 M ProtheraCytes^®^/well (+0.1 M PC and +0.25 M PC, respectively) in the absence (−INFL) or presence (+INFL) of inflammatory cytokines. Values are mean ± SD of measurements from 4 independent donors in 3 replicates. * = *p* < 0.05, ** = *p* < 0.01. ^#^ = *p* < 0.05, ^##^ = *p* < 0.01, difference between −INFL and +INFL at identical conditions.

**FIGURE 5:**
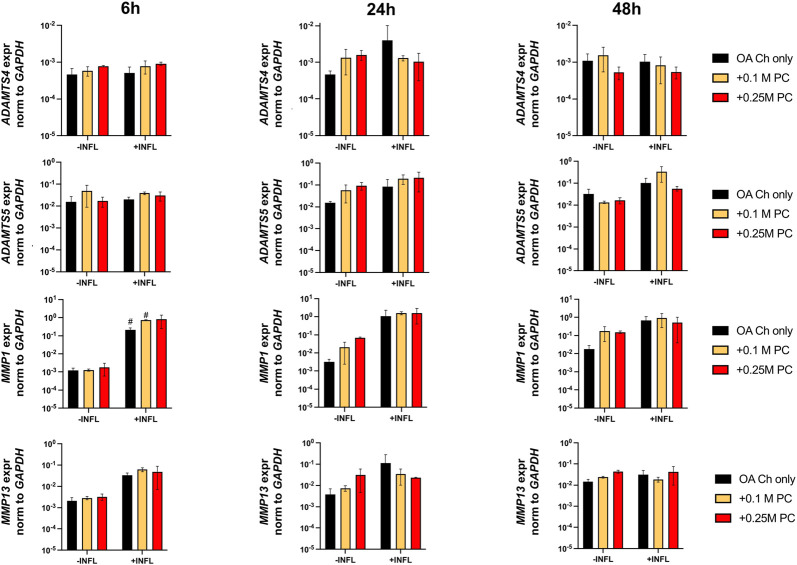
Expression levels of ECM-degrading genes in OA chondrocytes at different timepoints. Real-time reverse transcriptase–polymerase chain reaction of OA Chondrocytes after 6, 24 and 48 h of monoculture (OA Ch only) or co-culture with 0.1 or 0.25 M ProtheraCytes^®^/well (+0.1 M PC and +0.25 M PC, respectively) in the absence (−INFL) or presence (+INFL) of inflammatory cytokines. Values are mean ± SD of measurements from 4 independent donors in 3 replicates.

Gene expression analysis was performed to assess possible pro-anabolic or anti-inflammatory/catabolic effect of the ProtheraCytes^®^ on OA chondrocytes. We observed a decreased expression of collagen II (Col II) and increased expression of Col X when OA chondrocytes were exposed to inflammatory conditions (Figure 3). Aggrecan (ACAN) and Col II were significantly modulated by the presence of ProtheraCytes^®^ in the absence of inflammatory cytokines at different time points but no significant change was observed for the other chondrogenic genes (Figure 3). All the analysed pro-inflammatory (IL-1β, IL-6 and IL8/CXCL8) and the pro-degrading A Disintegrin and MetalloProteinase with Thrombospondin motifs 4 (ADAMTS-4), ADAMTS-5, Matrix MetalloProteinase-1 (MMP-1), and MMP-13 genes were more highly expressed in the inflammatory condition (INFL, Figures 4, 5). Instead, the expression of Nuclear Factor kappa light chain enhancer of activated B cells 1 (NF-kB1) was not modulated by INFL (Figure 4). ProtheraCytes^®^ showed a trend for the downregulation of the expression of IL-1β following 24 h of co-culture, but upregulated the expression of IL-8 at 48 h and the expression of IL-6 at 24 h of co-culture, but they did not modulate the expression of NF-kB1 (Figure 4). ProtheraCytes^®^ also showed a trend for the downregulation of the expression of ADAMTS-4 and MMP-13 at 24 h of co-culture in inflammatory conditions but no significant changes were observed in extracellular matrix (ECM)-degrading genes (Figure 5). Results from this first experiment showed that ProtheraCytes^®^ survived well when cocultured with OA chondrocytes in the presence of inflammatory cytokines and can modulate the expression of some inflammatory and pro-anabolic genes by the OA chondrocytes.

**FIGURE 6:**
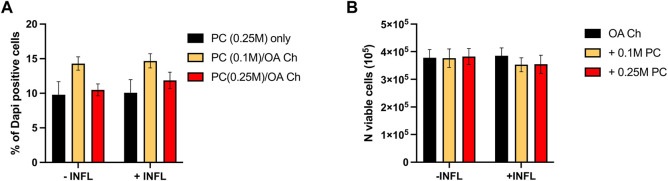
Assessment of ProtheraCytes^®^ and OA chondrocyte survival after 24 h. **(A)** Percentage of dead ProtheraCytes^®^ (PC) estimated cytoflurimetrically by evaluating the number of DAPI positive cells, after 24 h of monoculture [PC (0.25 M) only] or co-culture at 0.1 or 0.25 M cells/insert with OA chondrocytes [PC (0.1 M)/OA Ch and PC (0.25 M)/OA Ch, respectively] in the absence (−INFL) or presence (+INFL) of inflammatory cytokines. **(B)** Number of viable OA chondrocytes after 24 h of monoculture (OA Ch only) or co-culture with 0.1 or 0.25 M PC/well (+0.1 M PC and +0.25 M PC, respectively) in the absence (−INFL) or presence (+INFL) of inflammatory cytokines. Values are mean ± SD of measurements from 4 independent experiment with 3 replicates/experiment.

A second experiment was then performed with OA chondrocytes from three different donors (donors 2–4) cocultured for 24 h with two other batches of ProtheraCytes^®^ to corroborate the results obtained in the first experiment. We observed similar high viability of ProtheraCytes^®^ exposed or not to inflammatory conditions and slightly increased cell death in the lowest density group (0.1 M cells/mL) (Figure 6A). The number of viable OA chondrocytes did not significantly change when exposed to inflammatory conditions (Figure 6B).

**FIGURE 7:**
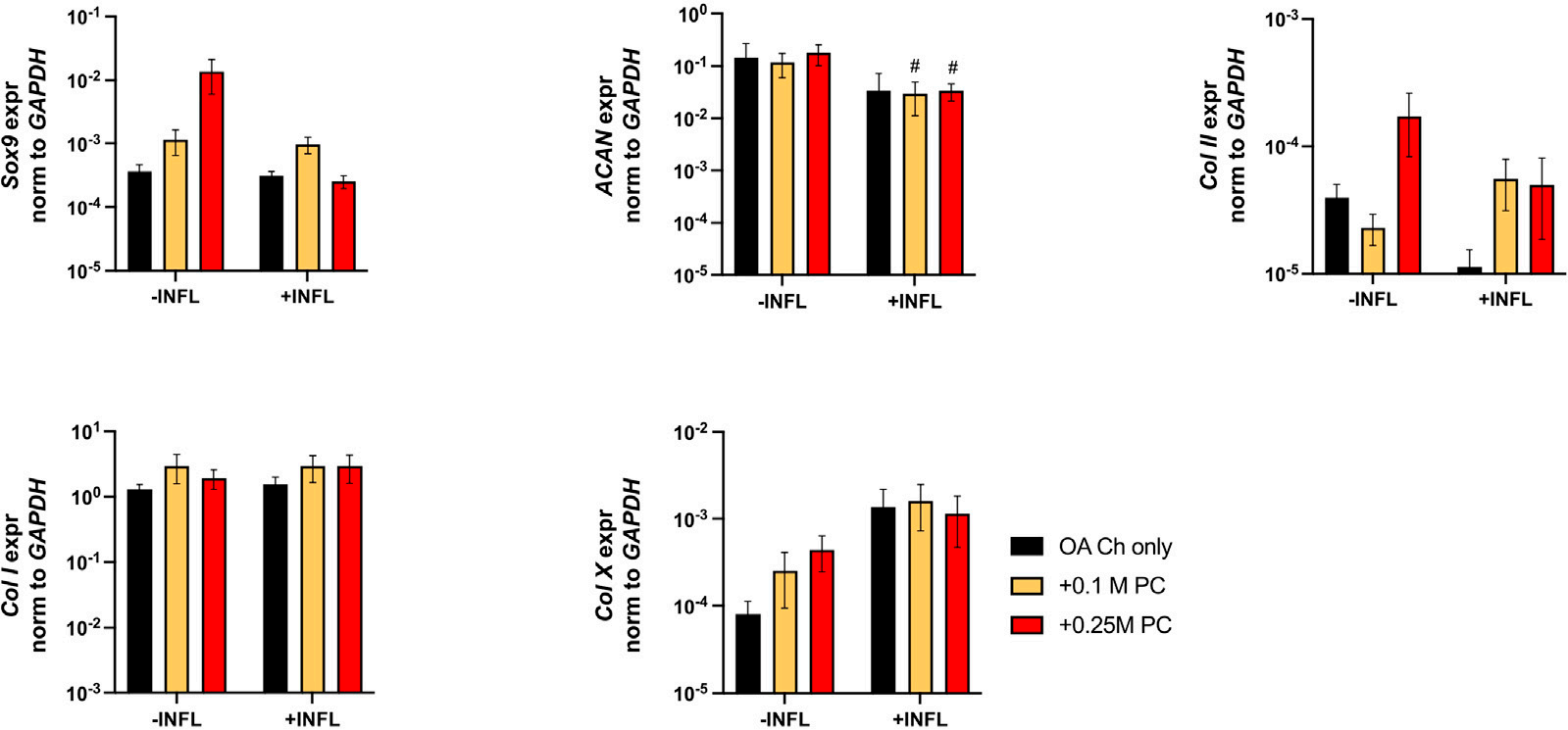
Expression levels of chondrogenic genes in OA chondrocytes after 24 h. Real-time reverse transcriptase–polymerase chain reaction of OA Chondrocytes after 24 h of monoculture (OA Ch only) or co-culture with 0.1 M or 0.25 M ProtheraCytes^®^ (PC)/well (+0.1 M PC and +0.25 M PC, respectively) in the absence (−INFL) or presence (+INFL) of inflammatory cytokines. Values are mean ± SD of measurements from 4 independent experiment with 3 replicates/experiment. Each replicate was analysed in duplicate.

**FIGURE 8:**
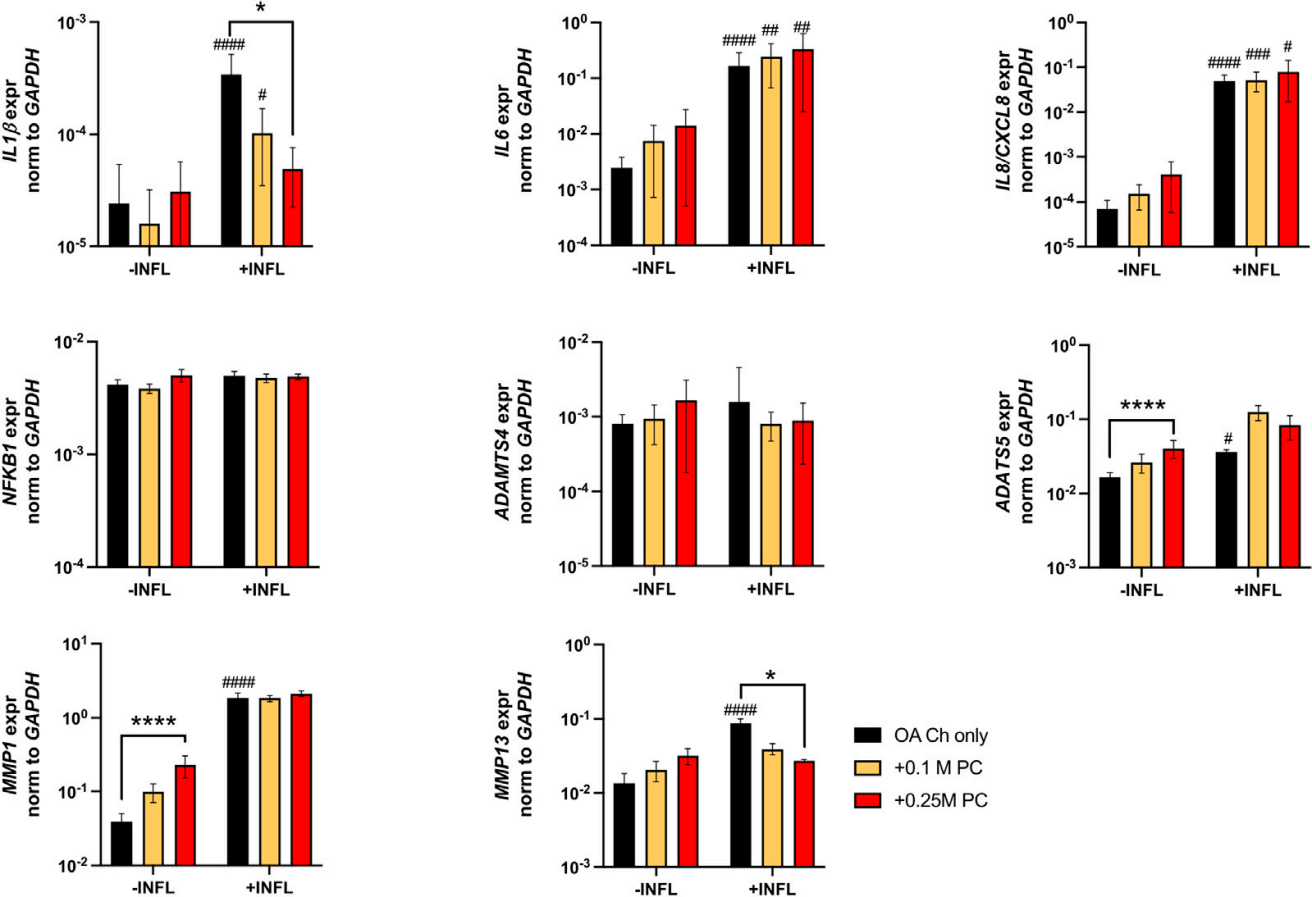
Expression levels of pro-inflammatory and ECM-degrading genes in OA chondrocytes after 24 h. Real-time reverse transcriptase–polymerase chain reaction of OA Chondrocytes after 6, 24 and 48 h of monoculture (OA Ch only) or co-culture with 0.1 or 0.25 M ProtheraCytes^®^/well (+0.1 M PC and +0.25 M PC, respectively) in the absence (−INFL) or presence (+INFL) of inflammatory cytokines. Values are mean ± SD of measurements from 4 independent donors in 3 replicates. * = *p* < 0.05, **** = *p* < 0.0001. ^#^ = *p* < 0.05, ^##^ = *p* < 0.01, ^###^ = *p* < 0.001, ^####^ = *p* < 0.0001 difference between −INFL and +INFL at identical conditions.

There was a reduction of ACAN and Col II expression and increase of Col X expression by OA chondrocytes when cultured in inflammatory conditions (Figure 7). We observed a trend of increased Sox9 and Col II expression when OA chondrocytes were cocultured with ProtheraCytes^®^ (Figure 7). As observed in the first experiment, the pro-inflammatory and pro-degrading genes were more highly expressed by OA chondrocytes in the inflammatory condition (Figure 8). ProtheraCytes^®^ at the higher density significantly downregulated the expression of IL1β and MMP13 by OA chondrocytes in the inflammatory condition but upregulated ADAMTS5 and MMP-1 in the non-inflammatory condition (Figure 8). ProtheraCytes^®^ did not modulate the expression of NF-kB1 by OA chondrocytes (Figure 8).

**FIGURE 9:**
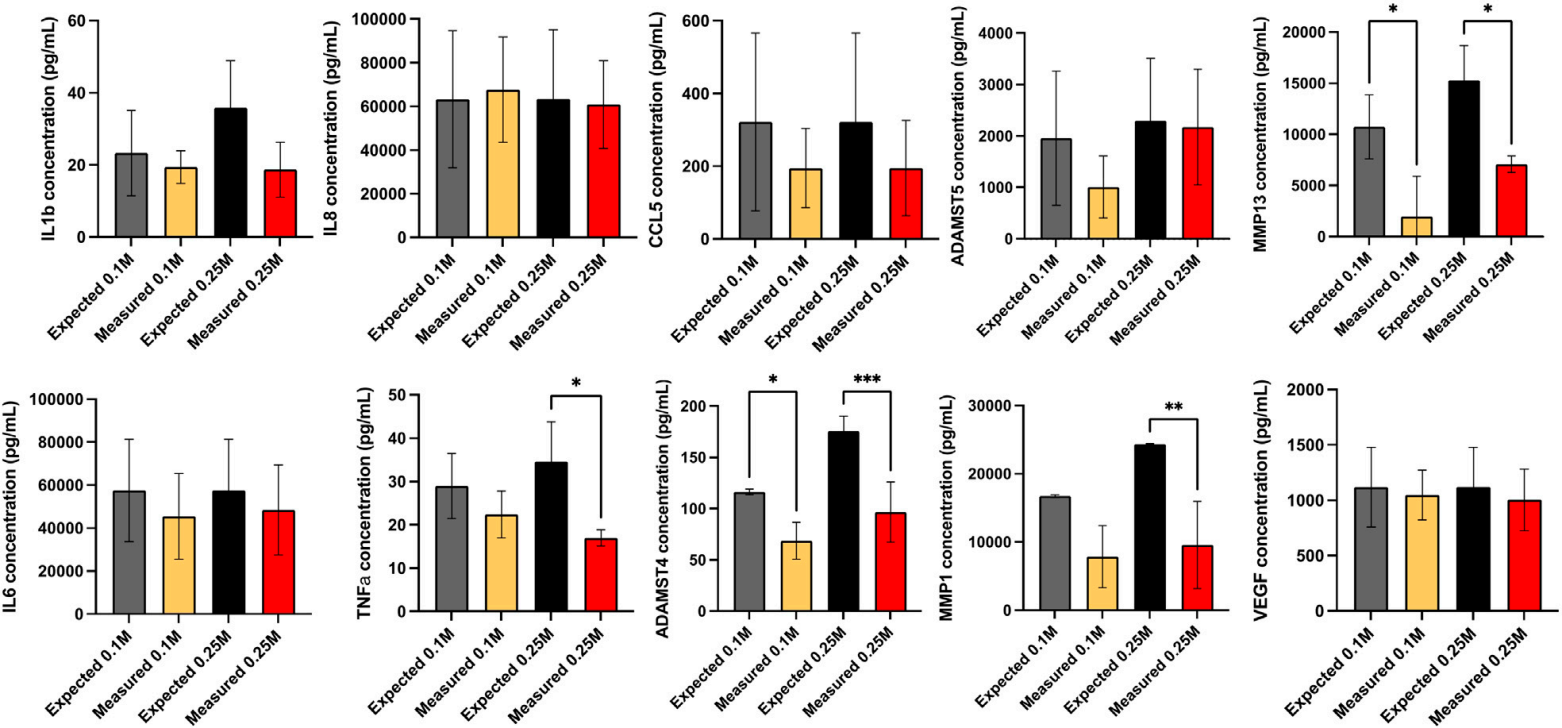
Secretome analysis after 24 h culture. Secretome of monocultured OA chondrocytes (OA Ch only), monocultured ProtheraCytes^®^ (PC only) and OA chondrocytes co-cultured with 0.1 or 0.25 M ProtheraCytes^®^/well in the presence (+IFLM) of inflammatory cytokines, quantified by multiplex immunoassays. The effect of factors secreted by ProtheraCytes^®^ on OA chondrocytes is displayed as the relative concentration (measured over expected concentrations). Values are mean ± SD of measurements from 3 independent experiment with 2 replicates/experiment. * = *p* < 0.05 difference, *** = *p* < 0.001 from OA Ch only.

The proteins secreted by OA chondrocytes when cocultured with ProtheraCytes^®^ were quantified in the supernatant by multiplex immunoassays. The release of the majority of the analysed proteins by OA chondrocytes was increased in the inflammatory conditions, with more than 10-fold increase in RANTES/CCL5, IL1β, IL6, and IL8/CXCL8 (Table 1). To assess the modulatory effects of ProtheraCytes^®^ on OA chondrocytes in the inflammatory condition, we compared the measured levels to the expected concentrations, calculated as the sum of the amount secreted by OA chondrocytes plus the amount defined in the OA chondrocytes/ProtheraCytes^®^ cocultures. Generally, ProtheraCytes^®^ at the highest dose (0.25 M cells/insert) had more pronounced anti-inflammatory/degrading effects on OA chondrocytes than the lower dose (0.1 M cells/insert) (Figure 9). In particular ProtheraCytes^®^ at the low dose significantly reduced the secretion of only ADAMTS-4 (1.6-fold) and MMP-13 (5.5-fold), whereas at the highest dose, significantly reduced the secretion of TNFα (1.9-fold), ADAMTS-4 (1.8-fold), MMP-1 (2.3-fold) and MMP-13 (2.0-fold) by OA chondrocytes (Figure 9).

**TABLE 1:** Secretome of monocultured OA chondrocytes (OA Ch only), monocultured ProtheraCytes^®^ (PC only) and OA chondrocytes co-cultured with 0.1 M [OA Ch/PC (0.1 M)] or 0.25 M ProtheraCytes^®^/well in the absence (−INFL) or presence (+INFL) of inflammatory cytokines, quantified by multiplex immunoassays.

	−INFL				+INFL			
	OA Ch only	PC (0.25 M) only	OA Ch/PC (0.1 M)	OA Ch/PC (0.25 M)	OA Ch only	PC (0.25 M) only	OA Ch/PC (0.1 M)	OA Ch/PC (0.25 M)
IL1β	0.37 ± 0.024	0.61 ± 0.05	0.49 ± 0.05	0.82 ± 0.09	18.2 ± 2.52^####^	17.2 ± 2.05	19.4 ± 1.26^#^	18.8 ± 2.2
IL6	292 ± 23.9	0 ± 0	305 ± 44.9	466 ± 83.6	5,763 ± 9,500^##^	0 ± 0	45,543 ± 5,266^#^	48,489 ± 5,786
IL8/CXCL8	136 ± 40.2	237 ± 29.1	147 ± 32.4	286 ± 43.6	59,490 ± 8,255^####^	346 ± 35.8	67,763 ± 8,168^####^	60,918 ± 6,212^##^
TNFα	0 ± 0	0 ± 0	0 ± 0	0 ± 0	22.4 ± 1.52^####^	10.1 ± 1.15	22.4 ± 1.52^####^	17.2 ± 0.94^#^
RANTES/CCL5	2.21 ± 1.28	0.06 ± 0.006	2.25 ± 1.08	3.36 ± 1.22	342 ± 139^###^	0 ± 0	195 ± 60.4^#^	195 ± 70.8^#^
ADAMTS4	124.7 ± 39.23	116.5 ± 34.8	79.61 ± 3.027	58.98 ± 14.6****	89.26 ± 13.41	90.32 ± 10.22	73.68 ± 11.91	96.46 ± 10.43
ADAMTS5	2,623 ± 1,036	576 ± 285.9	3,249 ± 1816	1,037 ± 747	1,699 ± 1,454	798.2 ± 321.8	2,983 ± 1,620	2,175 ± 1,144
MMP1	6,476 ± 1,619	4,275 ± 1950	185 ± 56.8	317 ± 140	11,649 ± 222	12,703 ± 196	7,866 ± 1,193	10,439 ± 1,568 ###
MMP13	2,422 ± 651	2,938 ± 1,111	2,866 ± 591	597 ± 185	7,734 ± 726 #	7,534 ± 726 #	1963 ± 1,025**	7,727 ± 304^####^
VEGF	774 ± 188	5.12 ± 1.27	708 ± 197	103.4 ± 18.62	1,120 ± 191	1.74 ± 0.39	1,049 ± 122	1,006 ± 155

Values are mean ± SD of measurements from 3 independent experiment with 2 replicates/experiment. * = *p* < 0.05, ** = *p* < 0.01, *** = *p* < 0.001, **** = *p* < 0.0001 difference from OA Ch only, and ^#^ = *p* < 0.05, ^##^ = *p* < 0.01, ^###^ = *p* < 0.001, ^####^ = *p* < 0.0001 difference between −INFL and +INFL at identical conditions defined by Mann-Whitney-U analyses.

### 3.3 Delivery with a needle for intra-articular injection does not impact ProtheraCytes^®^

**FIGURE 10:**
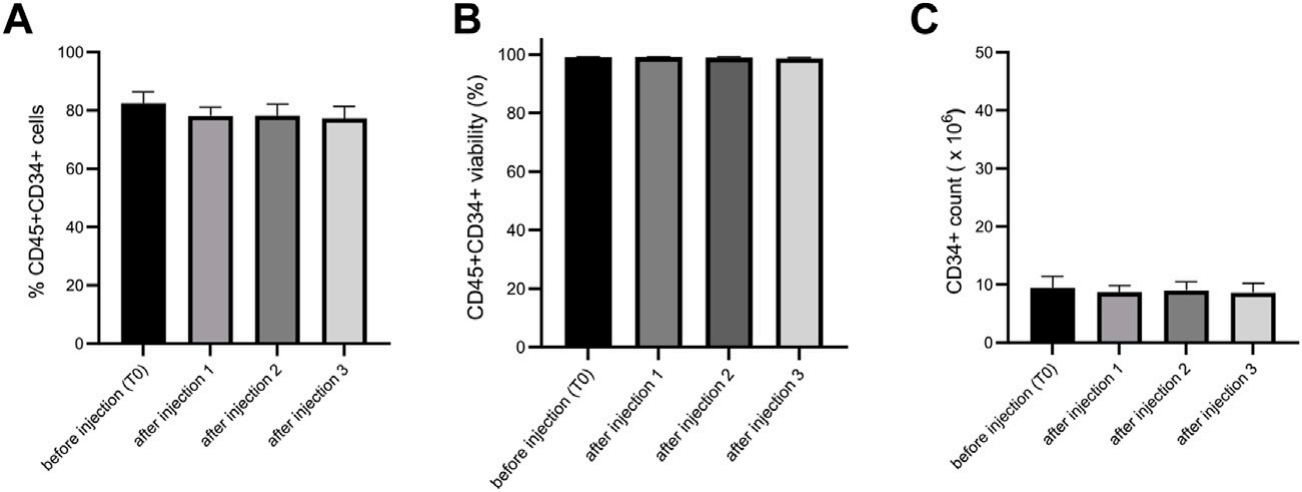
Analysis of the impact on ProtheraCytes^®^ after needle injection. The viability, purity, and CD34+ cell number of ProtheraCytes was analysed before and after injection through 20G needle and no significant differences were observed for any of the parameters. The same results were obtained with a 21G needle.

Before conducting preclinical studies, we wanted to test if the injection through a needle for intra-articular injection would alter the viability, purity, and CD34+ cell number of ProtheraCytes^®^. We tested two needle diameters for knee injection in humans and mice and showed no significant difference before and after injection for viability, CD34+ cell purity and number (Figure 10).

### 3.4 Biodistribution of ProtheraCytes^®^ after intra-articular injection in OA murine model

**FIGURE 11:**
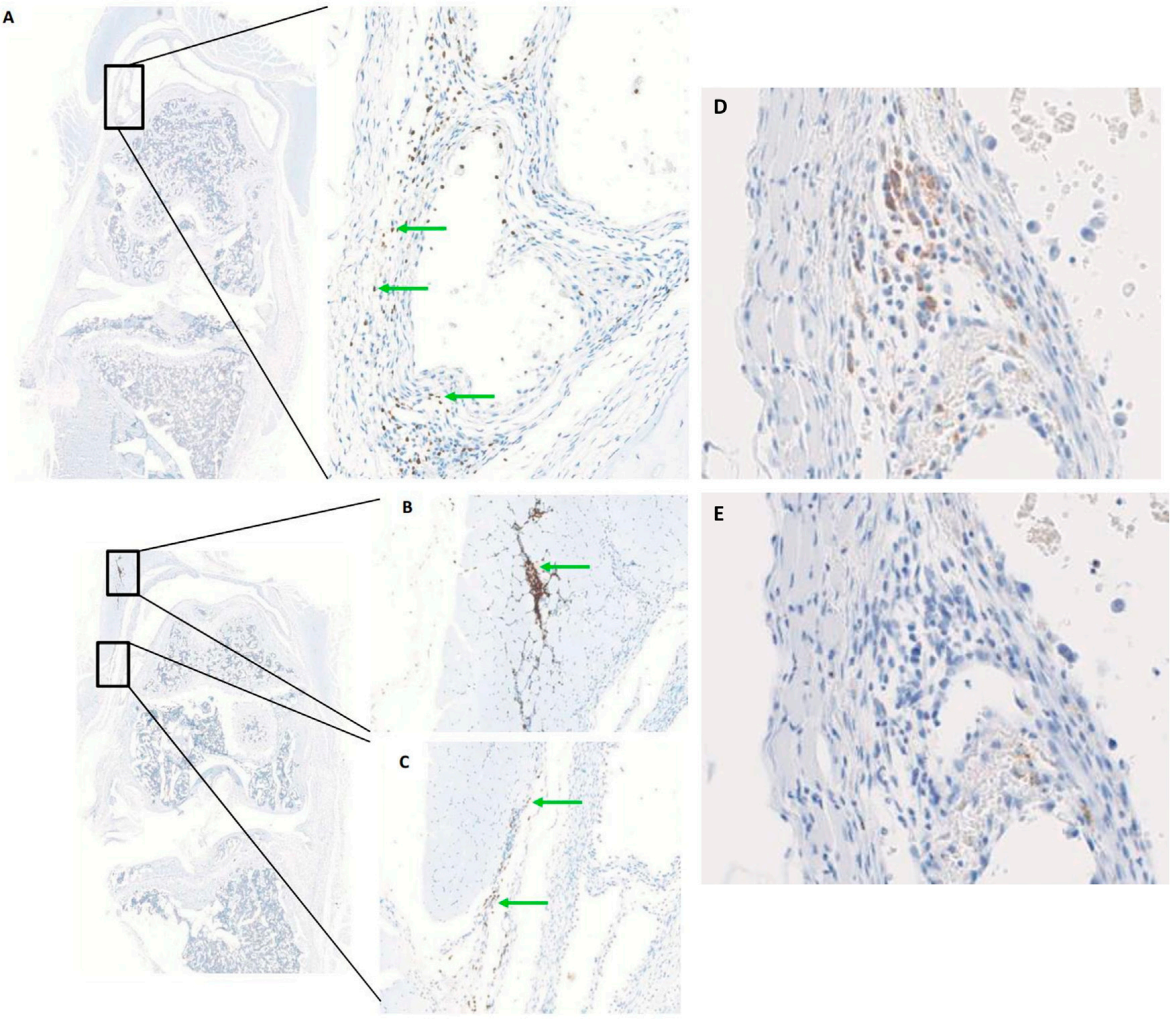
Biodistribution of ProtheraCytes^®^ after intra-articular administration 8 days after collagen-induced OA lesion. Representative histological sections of knee joints injected with ProtheraCytes^®^ at day 8 and sampled at day 9. Histological sections of knee joints showing the presence of a moderate number of ProtheraCytes^®^ in the synovial membrane **(A,C)** and muscle **(B)** (green arrows). Femurs are localized in the upper part and tibias on the lower part. Immunohistochemical staining of serial sections of the knee joint shown in **(A)** for the detection of human CD45^+^ **(D)** and apoptotic caspase 3^+^ **(E)** cells.

**FIGURE 12:**
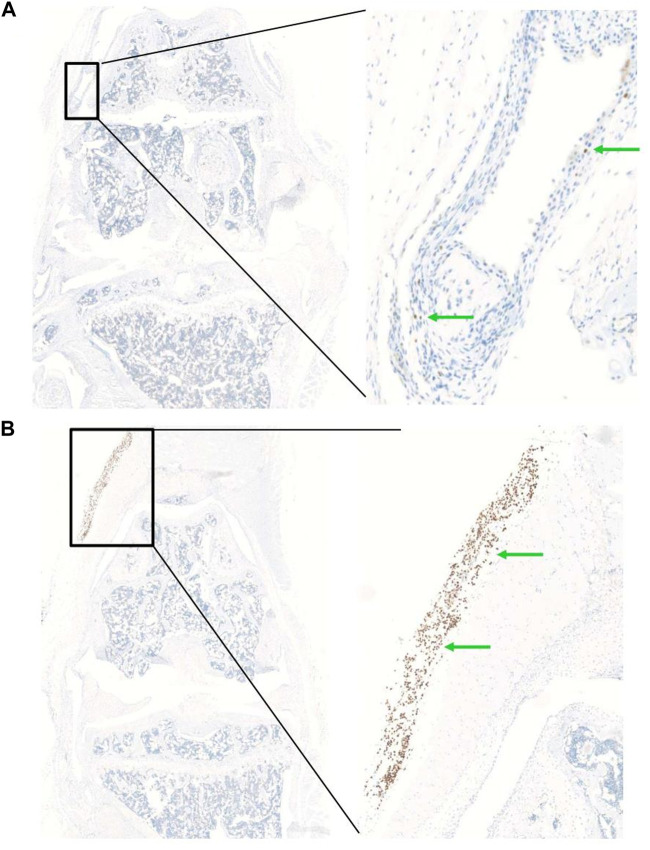
Biodistribution of ProtheraCytes^®^ after intra-articular administration 29 days after collagen-induced OA lesion. Representative histological sections of knee joints injected with ProtheraCytes^®^ at day 29 and sampled at day 30. Histological sections of knee joint showing the presence of a low number of human cells in the synovial membrane **(A)** and a high number of human cells in the muscle **(B)** (green arrows). Femurs are localized in the upper part and tibias on the lower.

In order to determine the optimal timing for cell injection, we performed a pilot study to evaluate the survival and biodistribution of ProtheraCytes^®^ after intra-articular injection in the knee joint of a collagenase-induced osteoarthritis (CIOA) immunodeficient mouse model. ProtheraCytes^®^ were injected at day 8 after CIOA and mice were euthanised at day 9, 15, and 29. We also injected ProtheraCytes^®^ at day 29 after CIOA and euthanised mice at day 30, 36, and 50. In the group of mice injected with ProtheraCytes^®^ at day 8, immunohistochemical analysis revealed the presence of a low to moderate number of human cells in 3/4 mice in each group sacrificed at day 9, 15 and 29. ProtheraCytes^®^ were located in the synovial membrane along the femur and the patella or in the muscle for one mouse sacrificed at day 9 (Figures 11A–C). Presence of CD45^+^ ProtheraCytes^®^ was not associated with caspase 3^+^ apoptotic signal indicating that ProtheraCytes^®^ survived after injection (Figures 11D, E). In the group of mice injected with ProtheraCytes^®^ at day 29, we observed the presence of low to moderate numbers of human cells in 2/4 mice sacrificed at day 30 and day 36 and no human cells were detected in animals sacrificed at day 50. For the animals euthanised at day 30 and 36, ProtheraCytes^®^ were detected in the synovial membrane and muscle (Figure 12).

## 4 Discussion

Osteoarthritis is the most common form of arthritis and a leading cause of disability worldwide, affecting millions of people (Litwic et al., 2013). OA is a degenerative joint disease that can lead to immobility, difficulty with daily activities, and disability (McDonough and Jette, 2010; Neogi, 2013; Clynes et al., 2019). OA is not simply a process of wear and tear but rather abnormal remodeling of joint tissues driven by a host of inflammatory mediators within the affected joint, resulting in pain, deformity and loss of function (Loeser et al., 2012). Unfortunately, there is no cure for OA but stem cell-based therapies have the potential to promote the repair of damaged joints by modulating the immune response, transplanting stem cell-derived chondrocytes, or stimulating the patient’s own cells for regeneration (Pers et al., 2018; 2016; Medvedeva et al., 2018). In this study, we wanted to evaluate the potential of ProtheraCytes^®^ for the treatment of OA.

First, we showed that synovial fluid from OA patients is not toxic to ProtheraCytes^®^ and maintains their viability when incubated for 96 h at 37°C, 5% CO_2_. It is possible that factors present in the synovial fluid such as hyaluronan and proteoglycan 4 (Tamer, 2013), might be responsible for the protection for ProtheraCytes^®^. This is encouraging data showing that synovial fluid would support the survival of ProtheraCytes^®^ if they were injected in the knee joint of OA patients.

We then evaluated the performance of ProtheraCytes^®^ using a relatively simple *in vitro* model consisting on their coculture with human OA chondrocytes in separate layers of Transwells, thus allowing communication between the two cell types through soluble factors. Such type of communication is supposed to occur if ProtheraCytes^®^ were injected in the synovial joint cavity of OA patients. To mimic the low-grade inflammatory milieu of the OA joint, we supplemented the culture medium with a cocktail of selected proinflammatory cytokines that have been described to play a pivotal role in OA (TNFα, IL1β, and IL6) (Mathiessen and Conaghan, 2017; Wang and He, 2018), at low concentrations (Acevedo Rua et al., 2021).

Clearly, the inflammatory condition used here exacerbated the inflammatory/degenerated traits of OA chondrocytes with the consequent upregulation of the expression of the inflammatory (RANTES/CCL5, IL1b, IL6, IL8/CXCL8) and degrading (ADAMTS-4 and-5, MMP-1 and -13) markers at protein and/or mRNA level. The inflammatory condition also promoted the downregulation of the expression of the cartilage genes collagen type II and aggrecan and the upregulation of the expression of the hypertrophic marker collagen type X.

We observed that ProtheraCytes^®^ remained highly viable once cultured for up to 48 h in such OA mimicking environment. Still, up to 20% of ProtheraCytes^®^ died upon coculture with OA chondrocytes. Lower cell mortality is expected to occur in a joint environment that is rich in synovial fluid. Indeed, based on the results of previous experiment, ProtheraCytes^®^ demonstrated high viability when exposed to synovial fluids from OA patients.

Importantly, our experiments demonstrated that ProtheraCytes^®^ reduce the expression of not only the pro-inflammatory factor TNFα by OA chondrocytes but also of key degenerative markers (ADAMTS-4, MMP-1 and MMP-13) known to play a significant role in aggrecan and collagen depletion in osteoarthritic cartilage (Malemud, 2019). Even if ProtheraCytes^®^ exerted more pronounced anti-inflammatory/degrading effects at the highest dose (0.25 M cells/insert), still, lower doses of ProtheraCytes^®^ (0.1 M cells/insert) induced OA chondrocytes to significantly reduce the expression of ADAMTS-4 and MMP-13. We showed that ProtheraCytes^®^ did not modulate the mRNA expression of NFkB1 mRNA in OA chondrocytes. Experiments to quantify the expression of additional molecules in the NFkB signalling pathway family as well as other factors belonging to other signalling pathway families known to modulate inflammatory responses in chondrocytes (i.e., JAK/STAT, MAPK) (Studer et al., 2004; Zhou et al., 2022), at protein and post-transcriptional levels, would be required to clarify the mechanisms responsible to some of the anti-inflammatory effects exerted by the ProtheraCytes^®^.

Unexpectedly, ProtheraCytes^®^ were observed to release relatively large amounts of ADAMTS-4, MMP-1 and MMP-13. We have, however, to consider that the rather artificial culture conditions used in our model could have caused the release of such factors by ProtheraCytes^®^. Future experiments using culture conditions more closely mimicking the joint environment should be considered to assess the secretome of ProtheraCytes^®^. Moreover, future studies should be performed to compare the immunomodulatory effects of ProtheraCytes^®^ to those of other cell types recently demonstrated to promote relieve of osteoarthritic symptoms such as adipose-derived mesenchymal stromal cells (Pers et al., 2016; Pers et al., 2018) and nasal chondrocytes (Acevedo Rua et al., 2021).

We investigated whether ProtheraCytes^®^ can induce pro-anabolic effects on OA chondrocytes. Our results showed that ProtheraCytes^®^ at the highest dose in the absence of inflammation induced OA chondrocytes to upregulate the expression of Sox9 and type II collagen. It is likely that ProtheraCytes^®^ induce these effects on OA chondrocytes via the secretion of paracrine factors, including exosomes containing anti-apoptotic miRNAs (21 and 146a) (Huang et al., 2016; Lu and Lu, 2020). Nevertheless, additional experiments using a model system more resembling the joint environment would be required to confirm the possible pro-chondrogenic effects of ProtheraCytes^®^.

We then evaluated the delivery of ProtheraCytes^®^ with the needle used for intra-articular injection to determine if it would modify the cells before conducting the *in vivo* studies. We observed that delivery through the intra-articular injection needle did not change the viability, CD34+ cell purity and number of ProtheraCytes^®^.

The survival and biodistribution of ProtheraCytes^®^ was then evaluated after intra-articular injection in the CIOA murine model in order to determine the best timing of administration in OA mice. Histological analysis of knee joints revealed that ProtheraCytes^®^ were detected in 75% of mice injected with cells at day 8 and sacrificed at days 9, 15, and 29. The number of detected human cells was higher in mice sacrificed at day 9 than at days 15 and 29 and the staining at day 9 was not associated with caspase 3^+^ apoptotic signal indicating that ProtheraCytes^®^ survived right after the injection. ProtheraCytes^®^ were always located in the synovial membrane no matter the day of mouse euthanasia, and we did not observe ProtheraCytes^®^ in the cartilage. This might be due to the fact that CD34+ cells are known to express CD44 (a receptor of hyaluronic acid, HA) (Legras et al., 1997) and thus can preferentially adhere to the synovial membrane, a tissue containing high amounts of HA (Revell et al., 1995) instead of cartilage or other joint tissues. Mesenchymal stromal cells (MSCs) also express CD44 and have been shown to home to the synovial membrane after injection in the knee joints of CIOA mice (Toupet et al., 2015). The synovial membrane plays an important role in maintaining tissue homeostasis within the intra-articular joint and producing the synovial fluid that nourishes the cartilage. The synovial membrane also constitutes a niche for MSCs (Kurth et al., 2011) and might be a favourable environment for exogenous MSCs and CD34+ cells.

When ProtheraCytes^®^ were injected at day 29 after CIOA, human cells were only found in 50% of the mice sacrificed at days 30 and 36 and in 0% of the mice sacrificed at day 50. This is in line with the results obtained when MSCs are injected in the knee joints of CIOA mice (Toupet et al., 2015). ProtheraCytes^®^ were mostly located in the synovial membrane and muscle. The muscle location might be attributed to the migration of cells but most likely to the difficulty of precise cell injection in the intra-articular space of the dislocated knee joints at day 29. Altogether, this study indicates that ProtheraCytes^®^ survive at least 20 days in the knee joint of CIOA immunodeficient mice when they are injected at an early stage of OA (day 9). Survival is limited to 7 days when cells are injected at day 29, when OA has progressed. These results are indicative of the survival of ProtheraCytes^®^ in an OA joint environment but must be interpreted with caution since only 4 mice were included in each group and 3 sections of the entire joint were examined. The limitations of this animal study include the low number of animals per group, the limited number of sections analysed per animal, and the lack of behavioural analysis. That is why these results need to be confirmed in larger preclinical studies that also evaluate the therapeutic efficacy of ProtheraCytes^®^ in this animal model.

## Institutional review board statement

Informed consent was obtained from all subjects involved in the study. The study was conducted according to the guidelines of the Declaration of Helsinki.

## Data Availability

The original contributions presented in the study are included in the article/Supplementary Material, further inquiries can be directed to the corresponding author.

## Supplementary material

SUPPLEMENTARY FIGURE S1 Representative Safranin-O image of a portion of a joint specimen from one donor considered in this study (donor 2, see *Material and methods* section). Scale bar 500 µm.
