# Supplementary figures and images for: Evaluation of expanded peripheral blood derived CD34+ cells for the treatment of moderate knee osteoarthritis
Source: Front Bioeng Biotechnol. 2023 May 23;11:1150522. doi: 10.3389/fbioe.2023.1150522 (PMC10242004; doi:10.3389/fbioe.2023.1150522)

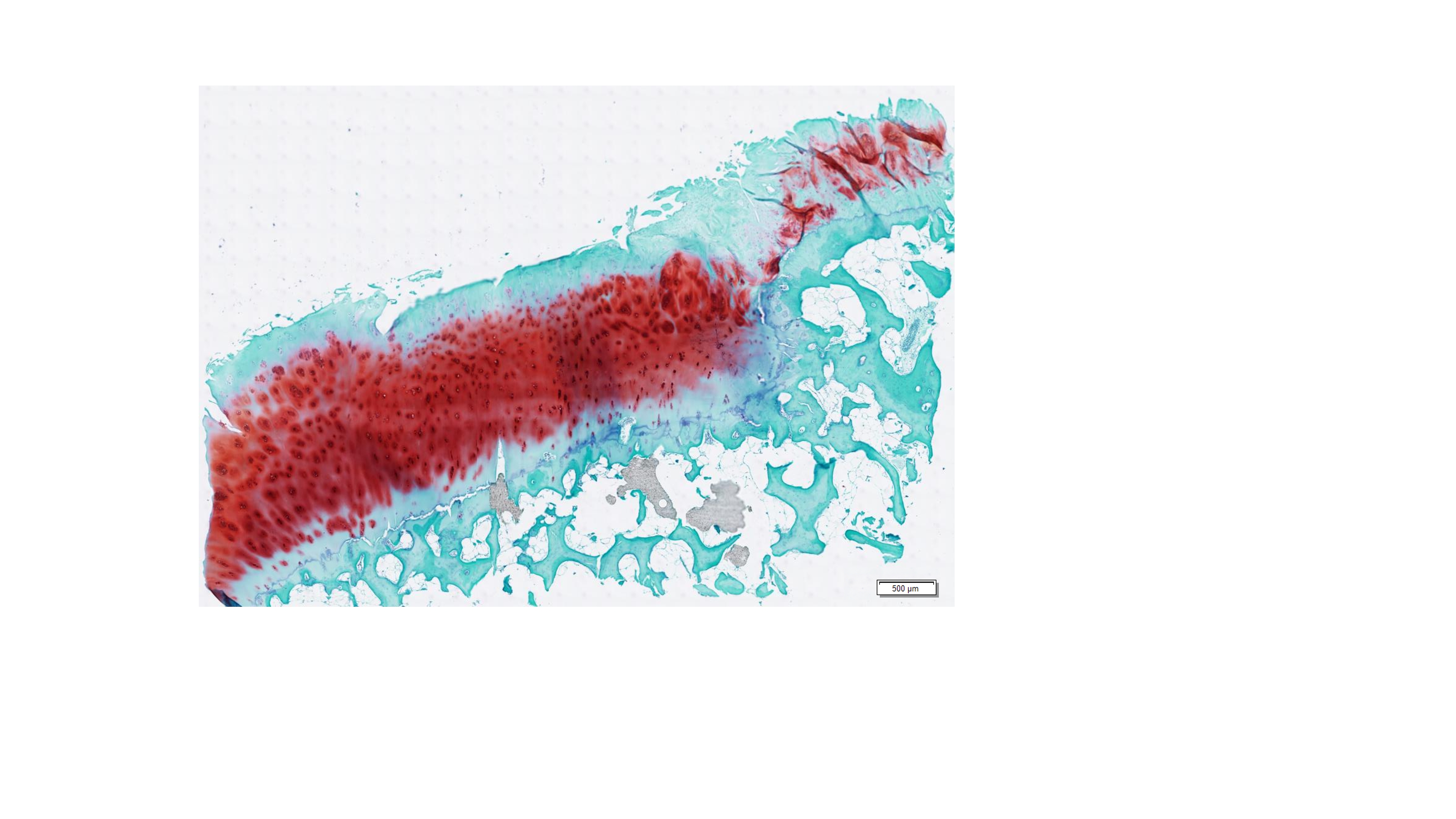

Supplement: Supplementary file 1 [file Image1.JPEG]
